# Plasma and cellular fibronectin: distinct and independent functions during tissue repair

**DOI:** 10.1186/1755-1536-4-21

**Published:** 2011-09-16

**Authors:** Wing S To, Kim S Midwood

**Affiliations:** 1 Department of Matrix Biology, Kennedy Institute of Rheumatology Division, Nuffield Department of Orthopedic Rheumatology and Musculoskeletal Sciences, Oxford University, 65 Aspenlea Road, London, W6 8LH, UK

## Abstract

Fibronectin (FN) is a ubiquitous extracellular matrix (ECM) glycoprotein that plays vital roles during tissue repair. The plasma form of FN circulates in the blood, and upon tissue injury, is incorporated into fibrin clots to exert effects on platelet function and to mediate hemostasis. Cellular FN is then synthesized and assembled by cells as they migrate into the clot to reconstitute damaged tissue. The assembly of FN into a complex three-dimensional matrix during physiological repair plays a key role not only as a structural scaffold, but also as a regulator of cell function during this stage of tissue repair. FN fibrillogenesis is a complex, stepwise process that is strictly regulated by a multitude of factors. During fibrosis, there is excessive deposition of ECM, of which FN is one of the major components. Aberrant FN-matrix assembly is a major contributing factor to the switch from normal tissue repair to misregulated fibrosis. Understanding the mechanisms involved in FN assembly and how these interplay with cellular, fibrotic and immune responses may reveal targets for the future development of therapies to regulate aberrant tissue-repair processes.

## Introduction

Fibronectin (FN) is a ubiquitous and essential component of the extracellular matrix (ECM). It functions both as a regulator of cellular processes and an important scaffolding protein to maintain and direct tissue organization and ECM composition.

During tissue repair, the body acts in a series of tightly regulated steps to rapidly reconstitute damaged tissue: the formation of a fibrin clot acts as the platform for granulation tissue assembly, with subsequent contraction and remodeling of the ECM to reform normal tissue [1,2]. Different forms of FN play differential and temporally discrete roles during tissue repair. Plasma FN and cellular FN possess distinct structures and rates of assembly into three-dimensional matrices.

In this paper, we discuss the differences in the structure of plasma and cellular FN, and their roles during the different stages of tissue repair. We summarize current theories of how FN is assembled into a three-dimensional matrix, and how this process is regulated. Understanding this complex matrix-assembly process may highlight potential targets for therapeutic advancement in the treatment of aberrant tissue-repair conditions.

### Structure of fibronectin isoforms

**Figure 1:**
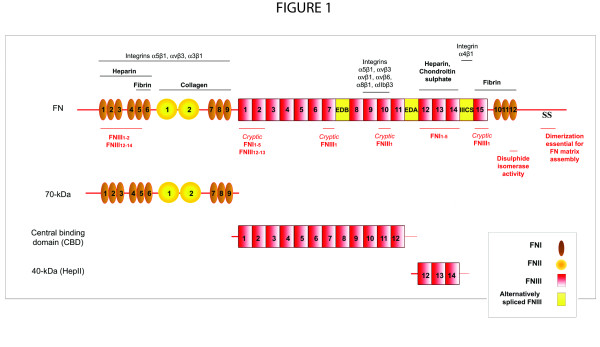
**Fibronectin (FN) and FN fragments**. FN is composed of a series of FNI repeats (dark-gray boxes), FNII repeats (circles), conserved FNIII repeats (light-gray boxes) and alternatively spliced FNIII repeats (EDA).

FN is a multi-domain glycoprotein composed of an array of multiple repeated modular structures: twelve FN type I repeats (FNI), two FN type II repeats (FNII), fifteen constitutively expressed and two alternatively spliced (in this paper, referred to as EIIIA and EIIIB) FN type III (FNIII) repeats, and a non-homologous variable (V) or type III connecting segment (IIICS) region. The multimodular structure and intermodular regions allow flexibility of the FN molecule, which is involved in regulating its function [3-8]. These modules are organized into functional domains, including the N-terminal 70-kDa domain (FNI_1-9_), the 120-kDa central binding domain (CBD; FNIII_1-12_) and the heparin-binding domain HepII (FNIII_12-14_). The specific domains of FN can interact with multiple binding partners, including other ECM components and cell-surface receptors [9]. FN is secreted as a dimer maintained by two disulfide S-S bonds at its C-terminus [7,10-12] (Figure 1).

#### Plasma fibronectin

Plasma FN is synthesized by hepatocytes and secreted into the blood plasma, where it circulates at 300-400 μg/ml [13] in a soluble, compact, inactive form. In soluble plasma FN, only one subunit possesses a V domain, and the EIIIB and EIIIA modules are absent [14-16]. Only very low levels (1.3-1.4 μg/ml) of FN possessing the EIIIA and/or EIIIB modules (cellular FN) have been reported to circulate in the blood plasma [17], but blood plasma levels have been shown to increase after major trauma resulting in vascular tissue damage, after inflammation, and in diseases such as atherosclerosis, ischaemic heart disease and stroke [18-21].

#### Cellular fibronectin

Cellular FN is synthesized by many cell types, including fibroblasts, endothelial cells, chondrocytes, synovial cells and myocytes [22]. Cellular FN is a mixture of FN isoforms. The alternative splicing of EIIIB and EIIIA and more complex splicing of the V or IIICS domain during transcription of cellular FN allows different isoforms of FN to be expressed in a tissue-dependent, temporally regulated, and cell-type-specific manner [15,23-26]. In humans, 20 potential FN isoforms can be generated [27]. Increased expression of the EIIIA+ and EIIIB+ isoforms of FN are associated with areas of physiological or pathological tissue remodeling, including wound healing and tissue repair. The observed isoforms of FN, and their association with physiological or pathological conditions, are outlined in Table 1. These isoforms modulate the properties of the ECM, and affect cellular processes.

**Table 1:** Cellular fibronectin (FN) isoforms reported during physiological and pathological conditions

FN isoform	Characteristics	**Ref**.
Physiological wound healing		
		
EIIIA	Expressed in tubular basement membrane by endothelium in rat model of acute renal failure; involved in regeneration of proximal tubules	[237]
	
	Increased expression by alveolar septal cells, albeolar macrophages and endothelial cells upon acute hyperoxic lung injury	[238]
	
	Absence results in abnormal wound healing in EIIIA^-/- ^mice	[90]
	
	Expressed in rat model of liver injury by sinusoidal endothelial cells	[98]

EIIIB	Increased levels in blood plasma after acute major trauma	[18]
	
	Increased expression by chondrocytes in muscularized arteries upon acute hyperoxic lung injury	[238]

EIIIA and EIIIB	Observed in granulation tissue by 7 days; EIIIB+ levels remain increased even after 14 days; EIIIA+ found around arterioles in connective tissue adjacent to the wound after 4 days	[95]
	
	Deposited in basement membrane zone of keratectomy wound models of corneal injury in rats	[239]
	
	Detected in ulcerated gastric tissue in rat models	[240]

EIIIA, EIIIB and V	All isoforms upregulated during rat corneal wound healing	[241]

Pathological conditions		

***Fibrosis***		

EIIIA	Idiopathic pulmonary fibrotic fibroblasts isolated from patients express higher levels of EIIIA+FN	[201]
	
	Involved in lung fibrogenesis in rat models of pulmonary fibrosis	[201]
	
	Highly expressed in mesangium and interstitium in rat glioblastoma multiforme and Habu snake venom models of renal fibrosis	[202]
	
	Increased expression in acute and chronic cutaneous graft-versus-host disease	[200]
	
	Increased in fibrotic periglomerular regions and areas of interstitial fibrosis	[26]
	
	Increased expression in human hepatic fibrosis	[242]
	
	Can induce the conversion of lipocytes to myofibroblasts; may play a role in hepatic fibrogenesis	[98]

EIIIB	Increased in obsolescent glomeruli	[26]

EIIIA and EIIIB	Increased in glomerulosclerotic lesions and fibrous crescents	[26]

Tumorigenesis		

EIIIA	Increased expression in hepatocellular carcinomas	[242]

EIIIB	Increased expression in interstitium and vascular intima of many primary human tumors including meningioma	[243]
	
	Expressed around neovasculature and stroma of many malignant head and neck tumors	[244]
	
	Detected around tumor stroma, tumor vasculature and in tissue adjacent to the invasion front of oral squamous cell carcinomas	[245]
	
	Detected in the stroma, in the cytoplasm of tumor cells and endothelial cells in the neovasculature of head and neck squamous cell carcinomas	[246]

EIIIA and EIIIB	Expressed in tumor blood vessels in mouse model of pancreatic tumorigenesis	[247]
	
	Present around the blood vessels of intratumoral microvessels in breast carcinomas.	[248]

Other		

EIIIA	Increased plasma levels in synovial fluid of rheumatoid arthritic joints	[249]

EIIIA, EIIIB and V	Increased expression in rat model of hypertension, especially of EIIIA+ form after 21 d	[250]

The orientation and rotational interdomain flexibilities of FNIII modules are known to be affected by neighboring domains, so the inclusion or exclusion of alternatively spliced domains may change the global conformation of FN, affecting the presentation of FNIII loop structures and binding sequences to modulate FN-cell signaling and FN-FN interactions during matrix assembly [9,28-33]. The structural composition of the different FN isoforms are important, as they play distinct roles in early and late wound-healing events [16].

### Fibronectin in early wound-healing responses

Plasma FN is a major component of the fibrin clot. Multiple mechanisms allow FN to be incorporated into the fibrin matrix. FN can interact via non-covalent interactions with fibrin via its FNI_1-5 _and FNI_10-12 _domains [34]. FN is also covalently crosslinked to fibrin by activation of the blood coagulation cascade involving activated Factor XIIIa (plasma transglutaminase or coagulation factor XIII) [2,14,16,35]. This crosslinks FN via glutamine residues within its N-terminus [36] to the fibrin α chains via ε-(γ-glutamyl) lysine cross links [37]. Furthermore, plasma FN can also be bound and then assembled into a high-molecular-weight multimeric matrix on the platelet surface. Platelet activation by thrombin induces increased cell-surface expression of the major platelet integrin αIIbβ3, which binds and assembles FN via a fibrin-independent mechanism [38-44]. Platelets also express a lower number of α5β1 and αvβ3 receptors on their surface, which mediate platelet adhesion [45]. Aggregated platelets can also assemble FN via a fibrin-dependent pathway [44]. The polymerization of fibrin into a three-dimensional network has been shown to be essential for FN assembly, as the γ chain of unprocessed fibrinogen signals via αIIbβ3 to inhibit this process [38].

**Figure 2:**
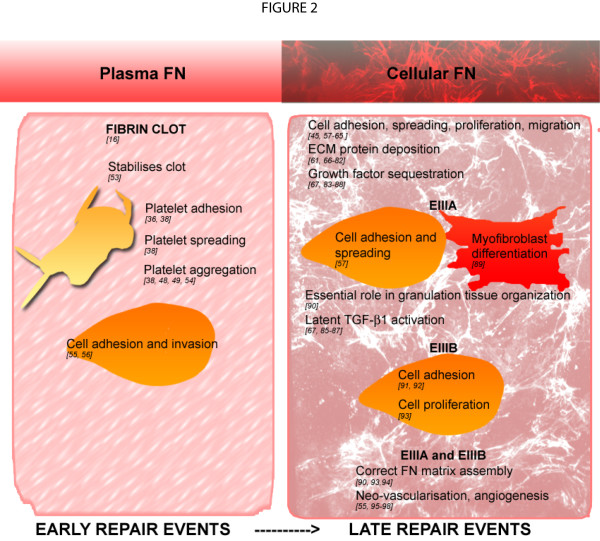
**Functions of plasma and cellular fibronectin (FN) during wound healing**. The different forms of FN play distinct roles during the different stages of wound healing.

Platelet-producing megakaryocytes endocytose and pinocytose FN from the plasma, which becomes packaged into the α granules of platelets [46]. This has been shown to involve the αIIbβ3 integrin; mice with a mutation in the gene encoding the fibrinogen γ chain (which prevents fibrinogen interactions with αIIbβ3), von Willebrand factor null mice, and fibrinogen null mice show increased FN levels in their α granules [47-49]. Upon platelet activation, FN is released from α granules in a process called degranulation. The FN released from platelets can also assemble on the surface of platelets [42,46,50]. FN incorporation into the fibrin matrix is important for various platelet functions, including adhesion, migration and aggregation (Figure 2) [16,36,38,45,46,48,49,51-98]. However, *in vitro *fibrin assembly was shown to be unaffected by a complete absence of plasma FN [51]. Furthermore, plasma FN conditional knockout mice, or mutant mice with reduced plasma FN levels of 50% or 70-80%, were shown to have normal clotting and bleeding times. Minimal effects on wound healing in these mice were seen *in vivo *[17,51,52]. Cellular FN isoforms possessing the alternatively spliced EIIIA and EIIIB domains, derived from platelets, are thought to compensate for the loss of plasma FN in these conditions [51]. Although it has been shown that cellular FN isoforms are not efficiently incorporated into the fibrin clots *in vitro*, it is thought that they may be sufficient to allow normal wound-healing events *in vivo*.

The fibrin-FN provisional matrix allows FN to adopt extended conformations within the fibrin-FN matrix, which leads to the exposure of cryptic cell binding domains to facilitate cellular processes (Figure 2) [36]. For example, fibroblast activation by various growth factors such as platelet-derived growth factor requires specific sequences within the heparin domain and IIICS region [99].

### Fibronectin in the late wound-healing responses

Endothelial cells and fibroblasts repopulate the wound and deposit cellular FN, an important and abundant component of granulation tissue [1,2,66,100]. FN organizes into fibrillar structures within the stroma of granulation tissue, and forms a dense network around fibroblasts, which polarize along the FN fibrils, parallel to the epidermis [66,95]. FN assembly into a three-dimensional fibrillar network on the cell surface is vital for establishing and maintaining tissue architecture and for regulating cellular processes including adhesion [57-60], spreading [101], proliferation [58,101-103], migration [99,104-106] and apoptosis [107,108] (Figure 2). The three-dimensional FN structural matrix plays an important role in regulating both ECM composition [61,67] and the deposition of other ECM molecules, including collagen types I and III [61,68-74], fibrinogen [75], fibrillins 1 and 2 [76-78], fibulin [79], laminin [61,73,80] and tenascin (TN)-C [81,82]. Reticulin has also been shown to colocalize with FN fibrils within the granulation tissue [66]. The FN matrix can also sequester growth factors and associated proteins, including bone morphogenetic protein-1 [83], vascular endothelial growth factor (VEGF) [84] and latent transforming growth factor (TGF)-β binding proteins (LTBP) 1, 3 and 4 [67,85-87] to regulate cell signaling events. While the FN matrix is continuously assembled, remodeled and turned over by cells, a more mature and stable ECM network assembles on this FN-matrix scaffold [61,85,88] (Figure 2).

FN in the wound site is also vital for regulating the neovascularisation of granulation tissue during the resolution of tissue injury. Exposure of different ECM protein conformations in the vascular basement during wounding acts as an important cue to regulate angiogenesis [55,96]. FN expression, especially of isoforms possessing the alternatively spliced EIIIA+ and EIIIB+ modules are highly upregulated around neovessels and capillary sprouts within the highly vascularized granulation tissue [95,97,98]. An FN fibrillar network has been shown to be required for proliferation and migration of human umbilical-vein endothelial cells in a three-dimensional environment [109], and functions to promote endothelial-cell survival [24,110] (Figure 2).

Myofibroblast differentiation is dependent on the presence of both TGF-β and EIIIA+FN and on the dramatic changes in the mechanical properties of the wound microenvironment [89,111,112]. Myofibroblasts form specialized actin-associated fibronexus adhesion complexes, which function in mechano-transduction to allow transmission of intracellular actin-generated contractile forces and the sampling of extracellular tension [111-114]. These specialized adhesion complexes may also function in myofibroblast-dependent contracture of the wound, which acts to 'shorten' and remodel the collagen-rich matrix, resulting in closure of the wound and recapitulation of normal tissue architecture and function.

The data highlighted here indicate distinct and discrete roles for the two different forms of FN during tissue injury and repair. Plasma FN and cellular FN are differentially expressed both temporally and spatially during wound healing: plasma FN circulates in the blood and functions during early wound-healing responses, whereas cellular FN is expressed and assembled locally and functions during later wound-healing responses. However, despite this non-overlapping expression and localization of the FN isoforms during wound healing, exogenous plasma FN can be assembled into pre-existing or newly assembling cellular FN matrices even if the plasma FN is isolated from a different species [115-117]. Although plasma FN shows slower initial kinetics of assembly than cellular FN [12], these data imply that plasma and cellular FN could potentially perform the same functions. Supporting this hypothesis, conditional plasma FN knockout mice were found to have normal wound healing and hemostasis [51], suggesting possible compensation by cellular isoforms of FN. However, in other physiological and pathological processes, these isoforms have been shown to be distinct and unique in their functions; plasma FN was found to be essential for protecting neuronal and non-neuronal cells from apoptosis after transient focal cerebral tissue ischaemia [51] and after traumatic brain injury [118], as cellular FN is not expressed in these damaged brain tissues. Furthermore, EIIIA-FN null mice were shown to have impaired abnormal skin wound-healing responses with reduced cell compaction and edematous-like areas within the granulation tissue and delayed re-epithelialization [90]. These results would suggest that EIIIA+FN plays an important role in the resolution of late wound-healing processes. Tan *et al*. reported that EIIIA-FN null mice on a different background strain showed no effect on wound healing, but did show reduced atherosclerosis, suggesting that cellular isoforms of FN contribute to pathological conditions [119]. These studies suggest independent roles for the different isoforms of FN, which cannot always be compensated for in their absence.

### Fibronectin-matrix assembly

There is still much that we do not know about how FN is assembled or how the rate of deposition is controlled. Understanding the mechanisms that regulate FN-matrix assembly will allow us to control this process when it becomes misregulated.

**Figure 3:**
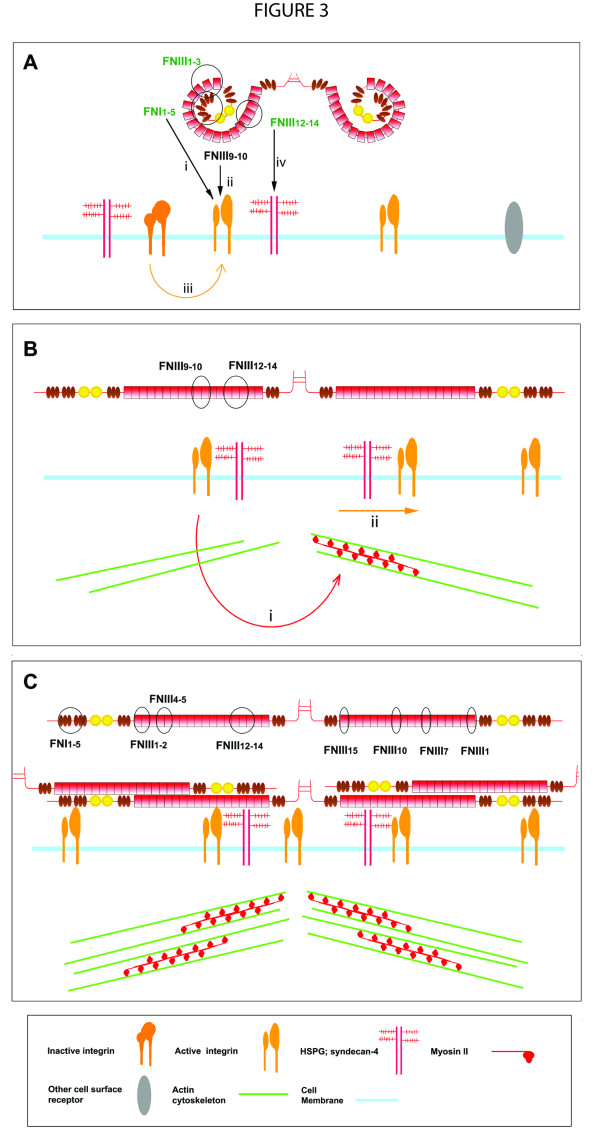
**Stages of fibronectin (FN)-matrix assembly: initiation, unfolding and fibrillar assembly**. (A) FN initiation involves interactions with cell-surface receptors: (i) FNI_1-5 _within the 70-kDa domain binds to cell-surface receptors possibly including integrins, (ii) FNIII_9-10 _binds to integrin α5β1, (iii) integrin activation by outside-in or inside-out signaling induces integrins to adopt a high-affinity state and allow FN binding and (iv) FNIII_12-14 _binds to heparan sulfate proteoglycans (HSPGs). (B) FN unfolding: (i) FN binding to cell-surface receptors induces cyctoskeletal reorganization of the actin cytoskeleton and myosin II-dependent contractility that results in (ii) receptor clustering and translation. This causes the tethered FN molecules to become unfolded. (C) Unfolding of FN results in the exposure of FN binding sites that allow FN-FN intermolecular interactions to occur. The domains important for each step are circled and denoted.

Plasma FN in solution alone will not polymerize [120] and will not form a three-dimensional matrix in the absence of cells [116]. Both plasma and cellular FN are expressed and secreted in a soluble, compact form, which is maintained by intramolecular electrostatic interactions between the FNI_1-5_, FNIII_1-2_, FNIII_2-3 _and FNIII_12-14 _domains [7,10-12] (Figure 3A). In low-salt conditions, this compact quaternary structure can be seen by electron microscopy [6,8], and Förster resonance energy transfer studies have shown that the arms of soluble FN overlap each other [121]. FN mutants lacking FNIII_12-14 _or having this region replaced by the alternatively spliced FN type III domains A1-A3 from tenascin-C (TN-C), had lower sedimentation coefficient values, reflecting adoption of a more open conformation and highlighting the importance of FNIII_12-14 _in maintaining these intramolecular interactions [7].

FN-matrix assembly is a stepwise, cell-mediated process [122,123]. The process seems to be rapid, as initial FN deposits appear at the cell surface within 10 minutes of plating, and in the conditioned medium within 30 minutes [122]. FN dimerisation is required for FN assembly, as mutation of C-terminal cysteines results in the loss of fibrillogenesis [124]. This process is briefly discussed below, but is discussed in more depth elsewhere [125].

#### Initiation of fibronectin-matrix assembly

Initiation of assembly involves FN binding to cell-surface receptors (Figure 3A(i, ii). Integrins that bind FN are summarized in Table 2, although some (for example, αvβ1) do not support FN fibrillogenesis. Mice engineered to be deficient in FN, α5, β1 or αv, or to express FN-RGE (FN^RGE/RGE^; the RGD integrin-binding sequence of FNIII_10 _is mutated to RGE) all exhibit embryonic lethality, highlighting the importance of FN fibrillogenesis and the FN fibrillar matrix during development [126-128].

**Table 2:** Fibronectin (FN)-binding integrins.

FN receptor	Supports fibrillogenesis	Domain	References
α3β1	+	70-kDa	[63,146]

α4β1	+	IIICS (V) CS1 region and EIIIA	[63,146]

α5β1	+	FNIII9-10 (RGD)	[22,60,143,144,251]

α9β1	-	EIIIA	[65]

α8β1	-	FNIII10 (RGD)	[63,64]

αvβ1	-	FNIII10 (RGD)	[63,64]

αvβ3	+	FNIII10 (RGD) or possibly 70-kDa	[139,143,144]

αvβ6	+	FNIII10 (RGD)	[63]

αIIbβ3	+	FNIII9-10 (RGD)	[38,63,145,252]

+ = Has been reported to be involved in FN fibrillogenesis.

- = Has been reported to be involved in adhesion to FN but not fibrillogenesis.

FN is thought to bind first to the cell surface via FNI_1-5 _within the 70-kDa N-terminal domain of FN (hereafter referred to as '70-kDa'; Figure 3Ai) [58,124,129-135]. Initiation of FN-matrix assembly occurs at focal contacts, which are initial sites of ECM contact on the cell periphery, which are rich in paxillin, vinculin, phosphotyrosines, and β1 and β3 integrins [136,137]. The receptor(s) for 70-kDa have not yet been clearly elucidated. Interactions with αvβ3 integrins have been shown to interact with the novel Gly-Asn-Gly-Arg-Gly (GNGRG) motif in FNI_1-5 _[126,138]. However, recent work has shown that mutation of the two GNGRG sequences within 70-kDa has no effect on the binding of 70-kDa or FN to adherent cells or on the ability of 70-kDa to compete for FN binding [139]. However, this study did show that interactions of cells with 70-kDa did require activated αvβ3 integrins [139], and it has also been shown that *in vivo*, the presence of pre-existing three-dimensional FN matrices also stimulates αvβ3 activation to induce FN-matrix assembly [116]. Another possible integrin-binding site is the Ile-Gly-Asp (IGD) sequence in FNI_9_[140]. It has been suggested that initiation of FN-matrix assembly may be a process that is not dependent on a single integrin or region within FN, but may involve many different molecules [125].

Other integrins may also be involved in the initiating the assembly of specific FN isoforms. In an interesting study, which demonstrated the essential role of FNIIIA+FN in lymphatic-valve development,α9β1 integrin was shown to be required for the assembly of EIIIA+FN by lymphatic endothelial cells [80]; however, this assembly mechanism may be isoform-specific, as a separate study found that overall FN-matrix assembly by EIIIA-FN null fibroblasts was unaffected [119].

It is well established that an essential step in the progression of FN-matrix assembly involves α5β1 integrin binding of the RGD loop on FNIII_10 _and the neighboring PHSRN sequence in FNIII_9 _in the CBD of FN [116,141-146] (Figure 3Aii). α5β1 integrin binding to CBD is a high-affinity interaction (Kd = 0.2 μmol/l by solid-phase binding) [28]. FN mutants with loss of the RGD site or a mutation within the RGD site were found to be unable to assemble a complex fibrillar network, forming only linear fibrillar arrays at the periphery of cells [12,139]. Antibodies against the RGD domain or small RGD peptides can also inhibit FN-matrix assembly [126,133,134,147,148]. Furthermore, FN^RGE/RGE^-expressing mouse embryos had normal FN distribution, and isolated embryonic fibroblasts could assemble short, thick, FN fibrils, confirming that FNIII_10 _interactions with α5β1 are not essential for assembly initiation [126].

The interaction of integrins with FN provides 'outside-in' signals [149], and induces integrin clustering in focal adhesions [60]. Ligation of integrins can also induce intracellular signaling and the activation of integrins to a higher-affinity binding state by 'inside-out' signaling. Phosphorylation of integrin cytoplasmic domains and subsequent integrin activation by signaling molecules such as talin requires myosin II-dependent cell-generated tension, which is activated by force-induced conformational changes [60,146,150,151]. 'Outside-in' signals can be translated into 'inside-out' signals via a feedback loop [141,149,151] to regulate adhesion-complex formation, integrin affinity and FN-matrix assembly [116,146,151] (Figure 3Aiii). 'Inside-out' signaling and integrin activation and clustering may precede FN binding during embryogenesis: integrin α5β1 activation and clustering was shown to be initiated by Eph/Ephrin signaling with subsequent FN-matrix assembly [152].

Cell-surface heparan sulfate proteoglycans (HSPGs) have also been shown to play an important role in FN-matrix assembly (Figure 3A (iv)) [153,154]. The HepII domain of FN (FNIII_12-14_) can interact with heparin [155], and the FNIII_13 _module is the primary binding site [156-158], suggesting that this FN domain is involved in the binding of HSPGs. Syndecan-2, which is the major syndecan of fibroblasts, has been shown to be an important HSPG for FN fibrillogenesis during zebrafish development [159], and supports FN-matrix assembly [160].

It should be noted that models of fibril initiation are often elucidated from two-dimensional cellular experiments, in which cells also form distinct adhesive structures. In three-dimensional cultures, cells that assemble FN do not form distinct focal or fibrillar adhesions, but instead form long, slender ECM attachments that contain both classic focal-adhesion and fibrillar-adhesion components: α5, paxillin, vinculin, focal-adhesion kinase, phosphotyrosine, and activated β1 [62]. These FN-assembly complexes probably involve a multitude of cell-surface receptors, HSPGs and signaling molecules that act in concert to facilitate FN-assembly initiation [125,161].

#### Fibronectin unfolding, elongation and fibril formation

Upon binding to integrins and other cell-surface receptors, FN then has to be unfolded from its compact structure into an extended structure (Figure 3B) [3,4,121].

An intact actin cytoskeleton is essential to generate cell tension and a cytoskeletal force, which translates into a temporal change in traction force that induces conformational changes of the cell-surface-bound FN and allows integrin crosslinking, clustering and subsequent translocation [4,60,121,136,162-164] (Figure 3Bi). FN unfolding is dependent on β1-FN translocation from focal contacts to central tensin-rich fibrillar-adhesion complexes [136] (Figure 3Bii), and FN fibrils are shown to form from the continuous extension of FN molecules and their intermolecular associations [136,148,162]. FN unfolding exposes binding sites buried in the soluble structure to promote the interaction of FN with other FN molecules and ECM components [165]. Cell contractility may require a submembranous pool of myosin II, controlled by myosin light chain kinase via RhoA-Rho kinase II-dependent [165-167] or RhoA-independent pathways [97,166,168]. Cells in three-dimensional microenvironments may favor different isoforms of myosin II for generating intracellular forces compared with cells cultured in two-dimensional environments [169]. The transduction of intracellular cytoskeleton-generated forces and extracellular mechanical stresses are communicated via cell-ECM contacts, allowing the cell to respond to changes in its microenvironment [161,170,171].

During embryogenesis, an alternative mechanism for production of intracellular tension involves the activation of the non-canonical Wnt/PCP signaling pathway, Rho GTPase Rac, p21 activated kinase (Pak) and cadherins, which generate cytoskeletal tension via cell-cell adhesion. This tension is transmitted via β1 to cell-surface-bound FN to mediate fibril formation [172]. In support of this process, inhibition of β1 function inhibits convergence extension tissue morphogenesis and cadherin-mediated cell adhesion [173]. The presence of multiple pathways to mediate cell contractility demonstrates the importance of this generated force in FN-matrix assembly.

FNI_1-5 _[58,124,174,175], FNIII_1-2 _[10,134,165,174,176], FNIII_4-5_[177] and FNIII_12-14_[132] are important domains for FN fibrillogenesis, and play a role in mediating and regulating intermolecular FN-FN interactions [12,124] (Figure 3C). The EIIIA or EIIIB domains can also promote FN-matrix assembly, possibly by exposing integrin-binding sites in neighboring domains: FNIII_10 _(EIIIB) and IIICS region (EIIIA) [94,178].

FN module unfolding and exposure of cryptic FN-FN binding sites further promote FN fibrillogenesis [121]. FN linker domains and the FNIII modules possess inherent elasticity [179-182]. Domains shown to possess cryptic binding sites include FNIII_1_[183-185], FNIII_1-2_[10], FNIII_10_[184], FNIII_7 _and FNIII_15 _[186] (Figure 3C). A peptide sequence derived from FNIII_1 _(FNIII_1_-C) can induce superfibronectin formation; that is, high-molecular-weight, crosslinked aggregates of FN, which resemble cell-assembled FN fibrils [185,187], indicating how effective these cryptic binding sequences are in promoting FN-FN interactions.

FN molecules are organized into thin fibrils of 5 nm in diameter, formed from the overlapping and staggering of extended FN dimers and the crosslinking of FN into stable multimers [69]. FN fibrils then become laterally associated into thicker fibrils of 6-22 nm in diameter [69]. Further FN-fibril interactions allow the formation of high-molecular-weight, complex, branched, fibrillar FN matrices, which are detergent-insoluble [122,123,129]. FN cross-linking and multimerization may occur via a partially cryptic endogenous protein disulfide isomerase activity present in FNI_12_, as reported by Lagenbach *et al*. in RNase refolding experiments [69,188]. However, there is some question as to whether covalent disulfide crosslinking is a true phenomenon; there are suggestions that FN associates via non-covalent protein-protein interactions [189,190]. The high-molecular-weight multimers seen in non-reduced SDS-PAGE gels are suggested to be a mixture of other ECM proteins such as fibrillin, which 'block' FN dimers from migrating through the gel [190]. This is not unexpected as FN is known to interact with many different ECM components, and these protein-protein interactions may well attenuate its migration through SDS-PAGE gels.

Once assembled, FN fibrils are continuously polymerized and remodeled within the fibrillar matrix on the cell surface [61]. FN remodeling is a dynamic process, in which fibrils continuously detach, contract, bend, stretch, extend, retract and anneal to neighboring fibrils [85,87,162,164,191,192]. *In vivo*, the fibrillar ECM structure is deformed by local cell migratory and protrusive activities [76,85,192,193], and large-scale tissue motion, particularly during embryogenesis [76,85]. This strongly extensible behavior is due to mechanical unfolding and refolding of FNIII modules [194].

Pre-existing three-dimensional matrices act as scaffolds for further FN deposition; new fibrils colocalize with pre-existing matrix [116,193,195]. Cells cultured in native ECM scaffolds deposit more soluble FN onto pre-existing FN fibers, requiring lower concentrations of FN for initiation [116,193]. New FN molecules deposited by cells onto a mature matrix are unfolded more than cells cultured on 'soft' polyacrylamide substrates [195]. ECM crosslinking has also been shown to increase the rate of *de novo *FN stretching by cells but to reduce overall deposition of soluble FN molecules [193,195], suggesting that ECM maturation is a mechanism that regulates the rate of FN deposition.

### Fibronectin and aberrant wound-healing conditions

FN-matrix assembly has to be tightly regulated. Cellular FN homeostasis is maintained by continual FN assembly and loss from the pericellular matrix [61]. Persistently high levels of FN promote cell proliferation, survival signals, and strong cell-ECM adhesions. Conversely, loss of FN-matrix assembly is observed in some transformed cells [168,196], and may be important during cell-migration events such as metastasis. In addition, loss of FN deposition [61,72] would accentuate the loss in the assembly of other ECM components within the wound bed, as seen in chronic non-healing wounds, even though FN expression persists and is actually increased in the surrounding dermis [197,198].

### Fibronectin in fibrotic conditions

FN plays an important role in the development of fibrotic disease [27]. Fibrosis is characterized by an excessive deposition of connective tissue that leads to the impairment of organ structure or function, and is considered a chronic inflammatory tissue-repair response, similar in structure and composition to granulation tissue [199,200]. In fibrosis, there is a key interplay between the immune response, fibroblastic-cell response and ECM, which results in the formation of fibrotic lesions.

Although collagen is the most predominant ECM component of fibrotic tissue, excessive deposition of FN also occurs, and precedes the collagen deposition (Table 1) [201-208]. In glomerular and interstitial fibrosis, there is markedly increased expression of total FN levels, with increased levels of EIIIA+, EIIIB+ and oncofetal (IIICS+) isoforms detected in distinct areas of the kidney and in areas of fibrosis [26,202-204]. Fibrogenesis is driven by fibroblasts and myofibroblasts, which show increased migration, proliferation, ECM synthesis and assembly within affected tissues [114,199,209]. The presence of both alternatively spliced EIIIA+FN and pro-inflammatory cytokines such as TGF-β1 have been shown to be required in the differentiation or transdifferentiation of cells to myofibroblasts [210]. For example, experiments with EIIIA null mice suggest that EIIIA+ isoform of FN induces α-smooth-muscle actin myofibroblast differentiation in the presence of TGF-β1. In the absence of EIIIA+ FN, there is continuous interstitial fibrosis after bleomycin treatment, but no switch to a chronic fibrotic response mediated by myofibroblasts. Furthermore, these experiments also showed that EIIIA+FN is required for latent TGF-β1 activation, and plays a response in fibroblast response to TGF-β1 [201,211]. TGF-β1 and the TGF-β family have been shown to activate the Smad family of transcription factors (including Smad3), which are involved in the expression of profibrotic genes [212]. Furthermore, mouse models of atherosclerosis have shown the essential role of FN in initima-media thickening *in vivo *[68]. Using a 49-residue sequence from the FUD domain of the F1 adhesin protein produced by *Streptococcus pyogenes *(pUR4), which has been shown to inhibit FN-matrix assembly by binding to the N-terminal 70-kDa domain of FN [213], this study demonstrated that inhibiting FN-matrix assembly *in vivo *significantly reduced intimal, medial and adventitial thickening, collagen deposition, cell proliferation, and inflammatory-cell infiltration after induction of atherosclerosis [68]. The results confirm the essential role FN plays in mediating ECM deposition and inflammatory response, which are corollaries of fibrosis. This study also showed promise for possible *in vivo *applications for inhibiting FN assembly.

Recently clinical studies have shown that use of a 585 nm flashlamp-pumped pulsed-dye laser resulted in the regression or arrest of keloid development by reducing the expression of TGF-β1in keloid tissues and increasing the expression of matrix metalloproteinase (MMP)-13 (also called collagenase-3) [214]. As many fibrotic conditions are largely untreatable, it is imperative that the mechanisms involved in the development and maintenance of these diseases are understood so that effective therapies can be developed.

### Regulation of fibronectin-matrix assembly

FN expression and assembly is stimulated in a cell-specific manner by a multitude of molecules (Table 3). Stimulation of FN expression, secretion and assembly by these agents emphasizes the complexity and requirement for tight regulation of this process. As mentioned earlier, TGF-β1 and connective tissue growth factor are key cytokines involved in upregulating FN expression during fibrogenesis [212,215,216]. FN deposition also requires RhoA-mediated cell contractility [4,121,165-167] (Table 3). FN can also be degraded by multiple proteases including MMP-9 [217]. FN fragments and modules can also inhibit FN-matrix assembly by competing for FN-assembly sites [187], which could act as a feedback system to regulate FN levels on the cell surface. The increased levels of proteases such as neutrophil elastase in chronic wound exudate, which can act to degrade FN [218-222], could further contribute to this condition. Aged clots in chronic wounds contain highly crosslinked fibrin, which is stripped of other functional proteins by the strongly proteolytic environment [223]. These examples illustrate the importance of FN and its assembly in regulating and resolving wound-healing processes to maintain tissue architecture.

**Table 3:** Regulators of fibronectin (FN) mRNA expression and assembly

	References
Positive regulators of FN mRNA	

TGF-β1 and the TGF-β family	[212,253-255]

Platelet-derived growth factor-BB	[256]

Insulin-like growth factor-1	[256]

Hepatocyte growth factor	[257]

Glucose	[258,259]

Glucocorticoids	[260]

Negative regulators of FN mRNA	

Cell contractility inhibitor	[3,121]

RhoA inhibitors	[165-167]

Positive regulators of FN assembly	

Sphingosine-1-phosphate	[261]

Estrogen	[262]

Plasminogen activator inhibitor type I	[263,264]

Urokinase plasminogen receptor	[265]

Connective tissue growth factor	[266]

Lipoprotein A	[165,167]

TGF = transforming growth factor.

FN fibrils are continuously remodeled and turned over, which is mediated via a β1-dependent, caveolin-1-dependent and low-density lipoprotein receptor-related protein (LRP)-independent endocytic mechanism [85,224]. FN becomes targeted to the lysosomes and degraded intracellularly [61,88]. As FN is assembled into high-molecular-weight multimers by an endogenous disulfide isomerase activity [188], some reverse proteolytic activity must occur to allow FN endocytosis [61,88]. Furthermore, β1 integrin clustering can induce polarized expression of membrane type 1 (MT1)-MMP to invasive structures to cause localized ECM degradation [225]. Indeed, endocytosis of fibrillar FN from pre-assembled matrices was shown to be much slower than endocytosis of soluble FN [224]. Interestingly, mature FN matrices are as highly dynamic as immature FN matrices, but ECM maturation has been reported to assemble less dynamic ECM networks over time, which loses this dynamic remodeling [85].

Other ECM components can also influence FN-matrix assembly. Low levels of vitronectin (VN) have been shown to enhance FN-matrix assembly by increasing the expression of matrix-assembly sites on the cell surface [136,226]. However, high concentrations of VN are inhibitory for FN-matrix assembly [226-228]. The HepII domain of VN has been shown to interact with αvβ3 and αvβ5 integrins, preventing actin microfilament reorganization and causing loss of FN-matrix assembly sites [228]. Loss of collagen type VI also impairs complex FN-matrix assembly; FN fibrils become oriented parallel to the long axis of the cell [229]. As discussed earlier, the individual FN domains including 70-kDa can also inhibit FN-matrix assembly by interfering with fibril formation by the full-length molecules.

Work carried out in our laboratory has also shown that smaller domains of TN-C, but not the full-length protein, can inhibit FN-matrix assembly [230,231]. As TN-C is only coexpressed with FN in areas of physiological and pathological tissue remodeling and the presence of encrypted inhibitory activity within the individual TN-C domains suggests TN-C may also play an important role in regulating FN-matrix assembly. This highlights how ECM composition and the breakdown of ECM components can also act as a further level of control, which could be exploited to control pathological wound-healing events.

### Future perspectives

Current therapies used to treat fibrotic conditions are well summarized and discussed elsewhere [232-234]. It is now appreciated that fibrosis can be considered an aberrant wound-healing response as the understanding of the mechanism of its development is better understood [234]. For example, anti-TGF-β has been successfully shown to reduce skin and pulmonary fibrosis in mice with sclerodermatous graft-versus-host disease, a mouse model of the systemic fibrotic condition scleroderma [235]. However, anti-TGF-β therapy has not been as successful in human systemic sclerosis (SSc): in a placebo-controlled phase I/II trial, systemic and repeated dosing of CAT-192, an antibody developed against active TGF-β1, showed no efficacy in a cohort of 45 patients with early-stage diffuse cutaneous SSc [236]. That study also had higher mortality rates than shown in trials of other drugs to treat diffuse cutaneous SSc, although whether this is due to other factors was not clear [236]. The lack of success might also have been due to the fact that other TGF-β isoforms have profibrotic effects [212], highlighting the complex interplay between the immune system, the ECM and cell signaling during wound healing and aberrant wounding responses. The interplay between cells and the ECM in the regulation of homeostasis and response to physiological and pathological events is complex, and it will be vital to understand these in order to develop therapies that can modify these processes.

Further research into the mechanisms that regulate FN-matrix assembly will help us understand how we can regulate it to prevent aberrant deposition that contributes to pathological conditions. In particular, the effect of other ECM proteins on the FN-assembly process may comprise a regulatory mechanism that could be further explored and exploited therapeutically. For example, ECM proteins such as TN-C are re-expressed only in tissues undergoing active remodeling, such as in fibrotic lesions. However, the role of these molecules in fibrotic tissues and their effects on FN expression, deposition or assembly are still unclear. Elucidation of the complex interplay between resident ECM constituents is likely to reveal how physiological, synergistic control of matrix remodeling is mediated.

## Conclusions

The plasma and cellular forms of FN play temporally and spatially distinct and vital roles during the progression of wound healing. Plasma FN circulates in the blood plasma in an inactive form, and is stored in the α-granules of platelets until activated by the wound response and stimulation of the coagulation cascade. Plasma FN is then deposited and crosslinked to the provisional FN-rich matrix, and functions to stimulate platelet adhesion and aggregation, and fibroblast spreading and invasion into the clot. Cellular FN is then synthesized by the migrated cells within the clot, and assembled into a complex, fibrillar matrix on the cell surface, which directs the deposition of other ECM proteins and the migration, adhesion and differentiation of fibroblasts. Many mechanisms are involved to regulate FN-matrix assembly, and there is now also growing evidence that in addition to regulation via molecules, the ECM composition and structure itself are also important.

As ECM assembly is such a complex process, understanding the mechanisms involved is vital if we are to manipulate this process. The fact that other ECM components can affect the deposition and assembly levels of FN suggests further levels of control that could be exploited in cases of dysfunctional wound-healing events. It may be that modifying the microenvironment by altering the expression of other ECM components may be sufficient to induce the resolution of such aberrant tissue-repair processes, which can lead to conditions such as fibrosis.

## List of abbreviations

ECM: extracellular matrix; EIIIA: alternatively spliced fibronectin type III repeat A; EIIIB: alternatively spliced fibronectin type III repeat B; FN: fibronectin; FNI: fibronectin type I repeat; FNII: fibronectin type II repeat; FNIII: fibronectin type III repeat; HSPG: heparan sulfate proteoglycan; MMP: matrix metalloproteinase; PDGF: platelet-derived growth factor; SSc: systemic sclerosis; TGF: transforming growth factor; VEGF: vascular endothelial growth factor.

## Competing interests

The authors declare that they have no competing interests.

## Authors' contributions

WST and KSM drafted the manuscript. Both authors have read and approved the final manuscript.
